# Synovium to Myocardium: A Case of Calcium Pyrophosphate Dihydrate Crystal Arthritis Associated With Myocardial Infarction

**DOI:** 10.7759/cureus.34528

**Published:** 2023-02-01

**Authors:** Mahanoor Raza, Masooma S Rana, Mobeena Arif, Taofeek Akinpelu, Abdul Waheed

**Affiliations:** 1 Family Medicine, WellSpan Good Samaritan Hospital, Lebanon, USA; 2 Family Medicine, Aga Khan University Hospital, Karachi, PAK; 3 Family Medicine Residency Program, WellSpan Good Samaritan Hospital, Lebanon, USA; 4 Family and Community Medicine, Penn State University College of Medicine, Milton S. Hershey Medical Center, Hershey, USA

**Keywords:** calcium pyrophosphate dihydrate crystal deposition, cppd arthritis, myocardial infarction, crystal arthropathy, pseudogout

## Abstract

Both gout and pseudogout are crystal-induced arthropathies. Here, we report a case of acute calcium pyrophosphate dihydrate (CPPD) arthritis associated with type 1 myocardial infarction (MI). An 83-year-old female presented to our emergency department with generalized weakness and bilateral lower extremity edema. Her left foot was noted to be more inflamed compared to the right, with cardinal signs of pain, swelling, erythema, and warmth. A presumptive diagnosis of cellulitis was made, and antibiotics were initiated. Further investigations revealed elevated troponins with new-onset bundle branch block, ST, and T-wave changes on electrocardiogram, indicating a type 1 MI. After a review of the patient’s history, imaging of the extremity, elevated inflammatory markers, and the typical distribution and pattern of inflammation, the diagnosis was changed to pseudogout. Steroids and colchicine were initiated, providing instant relief. This case highlights a possible association between cardiovascular disease and pseudogout, emphasizing the need for further studies regarding this relationship. Despite being rare, physicians should be made aware of this relationship, especially in patients with a history of CPPD arthritis presenting with type 1 MI.

## Introduction

Pseudogout is a crystal-induced arthropathy caused by calcium pyrophosphate dihydrate (CPPD) crystal deposition in the articular cartilage of joints. The prevalence of pseudogout has been reported to be 4-7% in the United States and Europe, with old age identified as a risk factor [1]. The deposition of CPPD crystals activates the innate immune system and triggers an inflammatory response, commonly presenting as inflammatory arthritis. Pseudogout may also manifest unusually and mimic other diseases, such as gout, osteoarthritis, and, as in this case, cellulitis. Maintenance of this pro-inflammatory state and subsequent dysfunction of vascular smooth muscle endothelium with oxidative stress can lead to plaque formation. Ultimately, acute rupture of the plaque causes myocardial infarction (MI) [2-4]. There have been reports on the relationship between gout, monosodium urate crystal deposition, and MI [4]. However, there is currently scarce discussion around the association between pseudogout and cardiovascular disease. To our knowledge, there are few papers in the literature reporting an increased risk for adverse cardiovascular events in patients with CPPD arthritis [5-7]. This is a report of acute CPPD arthritis associated with type 1 MI.

## Case presentation

An 83-year-old female was brought to the emergency room at WellSpan Good Samaritan Hospital on August 8, 2021, with complaints of bilateral leg swelling and pain. The onset of symptoms was two days ago. It was described as a burning, dull, aching pain which was worse on the left, rated as 8/10 on the pain scale, exacerbated with palpation and ambulation, non-radiating, and associated with a blister on the left foot.

The patient had a medical history of hypertension, type 2 diabetes with insulin use, mixed hyperlipidemia, glaucoma, left foot fracture and metal plate insertions, right nephrectomy in 2014 for renal cell carcinoma, anemia of chronic disease, parathyroidectomy for parathyroid adenoma in 2015, and open reduction and internal fixation of the right femur in 2020. The patient was also diagnosed with calcium pyrophosphate crystal deposition disease (CPPD) in April 2019 after discovering intracellular pyrophosphate crystals on fluid analysis of left ankle joint aspiration. However, the patient did not follow up with rheumatology and appropriate treatment was not sought. Her medications included amlodipine, metoprolol, hydralazine, lovastatin, and multiple eye drops. A review of the systems was positive for bilateral edema and negative for trauma, fevers, chills, chest pain, and shortness of breath.

**Figure 1:**
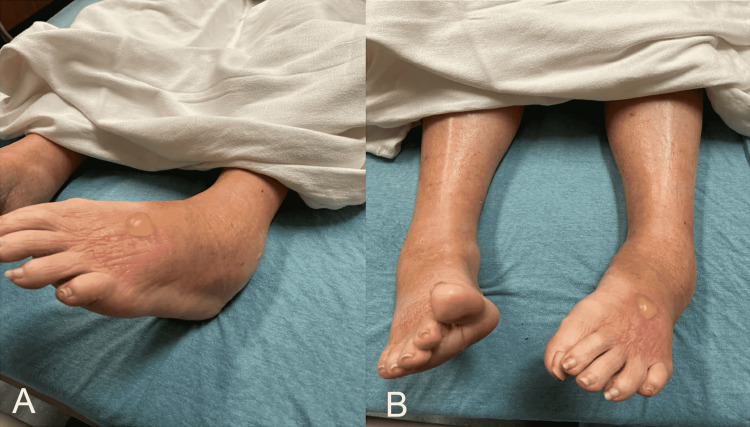
Lower extremity physical findings.

On arrival, the patient’s vital signs were blood pressure of 134/59, heart rate of 89 beats per minute, respiratory rate of 18 breaths per minute, a temperature of 37.7°C (99.9°F), and SaO_2_ of 96% on room air. Her physical examination was noticeable for an uncomfortable-appearing female with limited lower extremity motion secondary to pain, marked left ankle swelling with warmth and erythema, and a bulla on the dorsal aspect of the left foot (Figures 1A, 1B). Her sensation, strength, and distal pulses were intact and equal in bilateral lower and upper extremities. The differential was broad due to the unclear initial picture and complicated medical history. It included cellulitis, venous insufficiency, myxedema, nephrotic syndrome, deep venous thrombosis of the lower extremity, and pseudogout.

**Figure 2:**
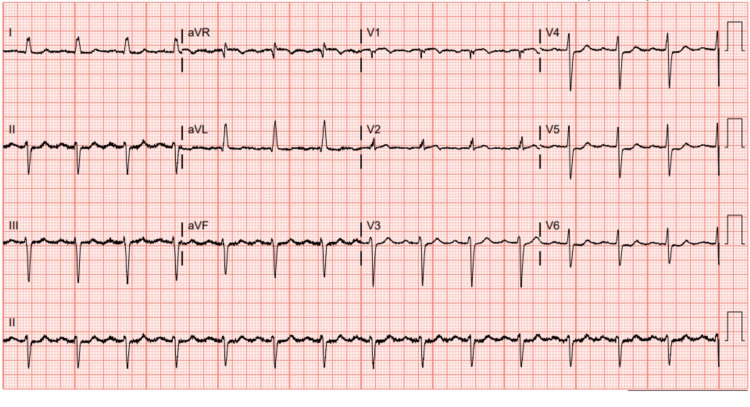
Electrocardiogram in sinus rhythm with left anterior fascicular block and non-specific ST and T-wave abnormality.

**Figure 3:**
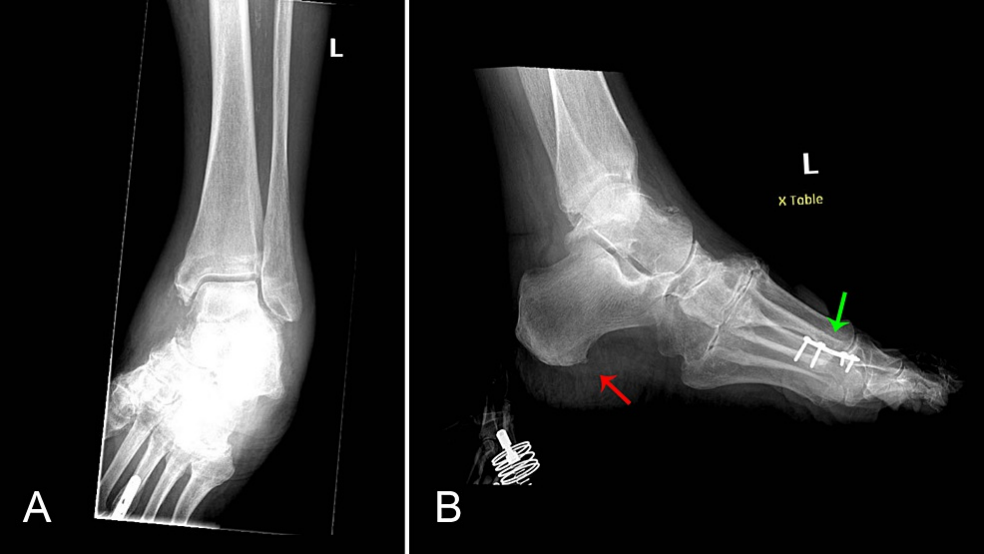
X-rays of the left foot (A) and left ankle (B) showing a metallic plate with screws along the distal diaphysis of the third metatarsal bone (green arrow), a small calcaneal spur (red arrow), some osteopenia, degenerative changes in tarsal metatarsal joints, and soft-tissue swelling.

Initial laboratory results were significant for troponin elevation of 0.04 ng/mL, which increased to 0.11 ng/mL (reference range: <0.03 ng/mL); B-type natriuretic peptide of 277 pg/mL, which increased to 380 pg/mL (reference range: <100 pg/mL), C-reactive protein of 134.9 mg/L (reference range: <10 mg/L); and sedimentation rate of 73 mm/hour (reference range: 0-30 m/hour). An electrocardiogram (EKG) showed a new left anterior fascicular block and new non-specific ST and T-wave abnormalities (Figure 2). A plain-film chest radiograph showed mild cardiomegaly. A bilateral lower extremity ultrasound was unremarkable. X-rays of the left foot and ankle showed a small calcaneal spur, degenerative changes in tarsal metatarsal joints, and soft-tissue swelling (Figures 3A, 3B).

She was initially treated with cefepime for cellulitis, but with a thorough chart review and discovery of the previous diagnosis of CPPD in her left ankle, antibiotics were stopped, and prednisone and colchicine were started. The patient noted instant relief in symptoms after this medication adjustment. Cardiology was consulted due to the elevated troponin and EKG changes. However, the patient was not considered a true acute coronary syndrome patient without the classic symptoms and was treated conservatively for type 1 versus type 2 MI with aspirin, metoprolol, and lovastatin. The patient was discharged on August 24, 2021, to acute rehab, with a prednisone taper and continued colchicine for pseudogout prophylaxis. The patient’s colchicine was discontinued after discharge from acute rehab.

**Figure 4:**
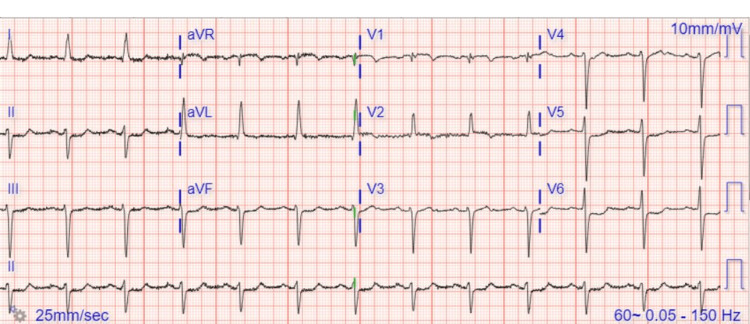
Electrocardiogram in sinus rhythm, with left anterior fascicular block and lateral ST-segment depressions.

**Figure 5:**
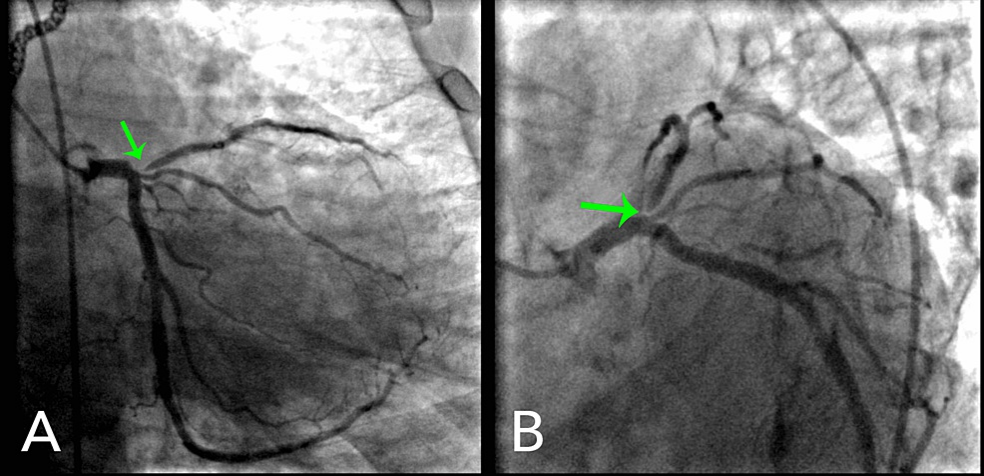
(A, B) Cardiac catheterization showing the left main coronary artery 20% occluded, left anterior descending ostium with 95% occlusion (green arrow), right-to-left collateralization, circumflex artery with diffuse mild stenosis, and right coronary artery 50% occluded at the ostium.

Interestingly, but unfortunately, the patient was readmitted to the hospital less than a month later with shortness of breath, EKG with increased prominence in ST changes, and moderate troponin elevation (Figure 4). Cardiac catheterization was performed on September 22, 2021, showing the left main coronary artery 20% occluded, the left anterior descending ostium with 95% occlusion, the right-to-left collateralization, the circumflex artery with diffuse mild stenosis, and the right coronary artery 50% occluded at the ostium (Figure 5). An echocardiogram showed new anterior wall hypokinesis (Video 1). This extensive coronary artery disease extension prompted immediate heparinization and coronary artery bypass graft procedure which was performed on September 29, 2021.

**Video 1:** Echocardiogram showing anterior wall hypokinesis.

## Discussion

This case highlights that CPPD can be challenging to diagnose in an acute setting and that there is a possible association between CPPD and type 1 MI. CPPD can have multiple presentations, ranging from asymptomatic to severe destructive polyarticular arthritis; therefore, it can mimic several diseases such as gout, osteoarthritis, and cellulitis. In our case, a presumptive diagnosis of cellulitis was made based on clinical suspicion. In any patient with features of acute joint swelling and signs of infection, such as erythema and tenderness, pseudogout should be considered a potential cause. Awan et al. also reported a case of CPPD deposition which led to chondrocalcinosis in the wrist joint, misdiagnosed as cellulitis [8]. There also have been multiple studies where pseudogout was mistaken for gout, osteoarthritis, rheumatoid arthritis, septic arthritis, Charcot arthropathy, or polymyalgia rheumatica [1,9,10]. It is essential that primary care clinicians consider pseudogout as a differential diagnosis when approached with a joint swelling presentation.

Chronic inflammation has long been linked with an increased risk of heart and vascular disease [11]. Moreover, acute cardiovascular events can be seen with many of these acute inflammatory conditions due to the development of demand ischemia causing type 2 MI, or an atherothrombotic plaque rupture causing type 1 MI [12-14]. Several studies have noted how vascular calcification and the release of interleukin-1 beta in acute CPP inflammatory arthritis may be associated with the formation of cardiovascular disease [1,15,16]. Inflammatory arthritis results in the suppression of nitric oxide, an increase in markers of oxidative stress, and the dysfunction of vascular smooth muscle endothelium, leading to plaque formation [17]. A retrospective cohort study among US Veterans with and without CPPD conducted by Bashir et al. noted common mechanisms that mediate both arterial and articular calcification, including deficiency of physiologic calcification inhibitors and upregulation of mediators of tissue injury, as well as increased major adverse cardiovascular events nearly three times higher in patients with CPPD when compared to controls. The incidence rate ratio for MI specifically was 2.93 (95% confidence interval: 2.52-3.40) [6]. Additionally, a recent cross-sectional study evaluating patients who underwent total knee arthroplasty found that CPPD patients had higher rates of in-hospital MI, especially those who were older than 80 years [5]. In our case, it may be true that our patient initially could have had just type 2 MI, which is more commonly seen and was managed medically, but she soon developed a clear presentation of type 1 MI as well. Our case has depicted how practicing physicians should be made cognizant of this relationship between CPPD crystal arthritis and type 1 MI so that appropriate management is initiated timely.

Cardiovascular disease is a leading cause of mortality in the United States, which stresses the importance of identifying risk factors and working on prevention [18]. Numerous studies have reported gout as an independent risk factor for MI and colchicine significantly lowering the risk of ischemic cardiovascular disease [4,19]. CPPD is a newly emerging risk factor for adverse cardiovascular events, and additional large-scale studies are warranted to explore this relationship further. CPPD treatment includes physical therapy, colchicine, non-steroidal anti-inflammatory drugs, and glucocorticoids; however, there are no treatments to prevent the formation of CPPD crystals other than treatment of the underlying condition predisposing to CPPD. Such modalities may be explored in the future, and their impact on cardiovascular events should be investigated.

Our patient was started on steroids and colchicine as soon as the CPPD diagnosis was established. It could be hypothesized that decreasing the inflammation played a role in the prevention of type 1 MI for as long as the patient took the medication; however, when it was discontinued, she returned with acute coronary syndrome. Inflammation plays a role in the pathophysiology of atherosclerosis, and there is a growing body of evidence regarding the use of low-dose colchicine in the prevention of cardiovascular disease in multiple clinical trials. The COLOT (Colchicine Cardiovascular Outcomes Trial) trial showed improved outcomes from the use of low-dose colchicine within one month of an MI, while the LoDoCo2 (Low-Dose Colchicine) trial reduced cardiovascular events in patients with stable cardiovascular disease taking low-dose colchicine [20]. To our knowledge, only one cohort study has explored this relationship between CPPD and cardiovascular disease [6]. Hence, further studies are necessary to shed more light on this association, as well as the importance of recognizing CPPD in acute settings and initiating preventative medication targeting both CPPD and cardiovascular disease.

## Conclusions

It is essential that primary care clinicians understand the underlying pathophysiology of CPPD and differentiate it from other forms of inflammatory arthritis and soft-tissue inflammation. This case indicates that clinicians must also keep a higher index of suspicion of acute cardiovascular disease with CPPD. Future studies may consider the effectiveness of colchicine for the prevention of both CPPD and cardiovascular disease. More large-scale studies are needed to further investigate this relationship.
